# The Role of Non-Typeable *Haemophilus influenzae* Biofilms in Chronic Obstructive Pulmonary Disease

**DOI:** 10.3389/fcimb.2021.720742

**Published:** 2021-08-04

**Authors:** Jake R. Weeks, Karl J. Staples, C. Mirella Spalluto, Alastair Watson, Tom M. A. Wilkinson

**Affiliations:** ^1^Clinical and Experimental Sciences, University of Southampton Faculty of Medicine, Southampton, United Kingdom; ^2^National Institute for Health Research (NIHR) Southampton Biomedical Research Centre, Southampton General Hospital, Southampton, United Kingdom; ^3^Birmingham Medical School, University of Birmingham, Birmingham, United Kingdom

**Keywords:** Non-typeable Haemophilus influenzae (NTHi), biofilms, chronic obstructive pulmonary disease (COPD), airways diseases, lung microbiome, host-pathogen interactions, antimicrobial tolerance

## Abstract

Non-typeable *Haemophilus influenzae* (NTHi) is an ubiquitous commensal-turned-pathogen that colonises the respiratory mucosa in airways diseases including Chronic Obstructive Pulmonary Disease (COPD). COPD is a progressive inflammatory syndrome of the lungs, encompassing chronic bronchitis that is characterised by mucus hypersecretion and impaired mucociliary clearance and creates a static, protective, humid, and nutrient-rich environment, with dysregulated mucosal immunity; a favourable environment for NTHi colonisation. Several recent large COPD cohort studies have reported NTHi as a significant and recurrent aetiological pathogen in acute exacerbations of COPD. NTHi proliferation has been associated with increased hospitalisation, disease severity, morbidity and significant lung microbiome shifts. However, some cohorts with patients at different severities of COPD do not report that NTHi is a significant aetiological pathogen in their COPD patients, indicating other obligate pathogens including *Moraxella catarrhalis, Streptococcus pneumoniae* and *Pseudomonas aeruginosa* as the cause. NTHi is an ubiquitous organism across healthy non-smokers, healthy smokers and COPD patients from childhood to adulthood, but it currently remains unclear why NTHi becomes pathogenic in only some cohorts of COPD patients, and what behaviours, interactions and adaptations are driving this susceptibility. There is emerging evidence that biofilm-phase NTHi may play a significant role in COPD. NTHi displays many hallmarks of the biofilm lifestyle and expresses key biofilm formation-promoting genes. These include the autoinducer-mediated quorum sensing system, epithelial- and mucus-binding adhesins and expression of a protective, self-produced polymeric substance matrix. These NTHi biofilms exhibit extreme tolerance to antimicrobial treatments and the immune system as well as expressing synergistic interspecific interactions with other lung pathogens including *S. pneumoniae* and *M. catarrhalis*. Whilst the majority of our understanding surrounding NTHi as a biofilm arises from otitis media or *in-vitro* bacterial monoculture models, the role of NTHi biofilms in the COPD lung is now being studied. This review explores the evidence for the existence of NTHi biofilms and their impact in the COPD lung. Understanding the nature of chronic and recurrent NTHi infections in acute exacerbations of COPD could have important implications for clinical treatment and identification of novel bactericidal targets.

## Introduction

### Chronic Obstructive Pulmonary Disease

COPD is currently the third leading cause of global mortality affecting an estimated 174 – 384 million people worldwide and is responsible for 3.2 million deaths annually (Rabe and Watz, 2017). COPD is caused by the inhalation of noxious particles and gases and at least 90% of COPD patients live in developing countries where the disease is mainly associated with biomass fuel burning and pollution. The remaining 10% live in developed countries where the predominant cause is cigarette smoking (Rabe and Watz, 2017). COPD is a heterogenous progressive, inflammatory syndrome of the lungs, characterised by the co-existence of predominantly two distinct syndromes; chronic bronchitis and emphysema (Rabe et al., 2007). Together these conditions result in a chronic productive cough, poorly reversible airflow obstruction and alveolar parenchymal destruction (MacNee, 2006), punctuated by episodic acute exacerbations of COPD (AECOPD), that lead to hospitalisation (Wilkinson et al., 2017).

Chronic bronchitis is particularly important with respect to the role of bacteria and chronic infections. An immunological cascade in response to inhaled particles and gases drives airway structural remodelling that leads to hypertrophy and hyperplasia of sub-mucosal bronchial glands and mucus-producing goblet cells (MacNee, 2006), forming a viscous and alkaline mucus layer to trap particles and bacteria. Excess mucus production and damage to the airway epithelium by inflammation and oxidative stress results in ciliary dysfunction, which is also caused by the direct effects of smoking. This impairs mucociliary clearance and leads to the formation of obstructive mucus plugs, changing the biotic and abiotic conditions of the lung (McDaniel et al., 2015; Panchabhai et al., 2016). Static mucus provides a nutrient-rich substrate for the colonisation of bacteria and mucus plugs trapped in bronchioles may form local anaerobic regions (McDaniel et al., 2015). Further immunological responses to bacterial infection include the formation of neutrophil extracellular traps (NETs) in severe COPD patients that has been associated with more frequent exacerbations and a reduction in lung microbiome diversity (Dicker et al., 2018), as well as modulation of the complement system that enhances phagocytotic clearance of bacteria (Ahearn et al., 2017).

Together these conditions may lead to significant microbiome shifts and favour the proliferation of fastidious, facultatively anaerobic pathogens, and drive a biofilm lifestyle.

### The Lung Microbiota in Airways Diseases and COPD

Contrary to historical views, the lung is not a sterile environment but has a rich and diverse microflora. The role of the lung microbiome and microbial dysbiosis in respiratory diseases is becoming increasingly understood through the advent of 16S rRNA sequencing of sputum, deep lung brushes and biopsy samples (Pragman et al., 2016), where the microbial composition of sputum samples closely resembles that of the low biomass bronchial and peripheral lung samples (Pragman et al., 2018). This has led to the characterisation of microbial patterns of respiratory disease and of COPD sub-types (Mayhew et al., 2018), supporting a patient-specific approach to treatment. However, there is currently no formal curated database of the Human Lung Microbiota, which has not formed part of the Human Microbiome Project (Beck et al., 2012). Therefore, there is disparity across lung microbiome investigations (**Table 1**) into airways diseases, partly driven by the lack of standardisation of methods of sample acquisition and processing (Pragman et al., 2018).

**Table 1:** Summary of Key Studies Investigating AECOPD Microbiology.

Finding	Main Result(s)	Citation
NTHi proliferations cause AECOPD leading to hospitalisation.	NTHi was detected in the sputum of 70% patients in a large cohort study of 105 moderate (45%) and severe (40%) COPD patients (GOLD stage 2 - 3). During AECOPD, culture and PCR analysis revealed NTHi populations significantly proliferate compared to baseline.	(Wilkinson et al., 2017)
*Streptococcus* spp. *and M. catarrhalis* contribute to AECOPD.	Culture and PCR analysis of sputum revealed *Streptococcus pneumoniae* remained a dominant lung taxon during AECOPD (but proportions did decrease). *M. catarrhalis* proliferated during AECOPD.	(Wilkinson et al., 2017)
Viral interactions contribute to AECOPD and drive NTHi proliferation.	PCR showed increased co-infection of NTHi and HRV between exacerbation-state (29.2%) and stable state (9.1%) patients, with 46.5% patients having at least one HRV-positive exacerbation.	(Wilkinson et al., 2017)
	Inoculation of 14 mild COPD (GOLD stage 2) patients with human rhinovirus (HRV) resulted in a 21% *Haemophilus* spp. proliferation driving a persistent infection. NTHi was detected in sputum by 16S rRNA pyrosequencing using the V3-V5 16S hypervariable regions.	(Molyneaux et al., 2013)
NTHi proliferation is associated with AECOPD severity.	Microbiome analysis of 584 sputum samples from a cohort of 101 COPD patients with moderate (44.6%), severe (39.6) to very severe (15.8%) COPD (GOLD stage 2 – 4) showed that there was a significant increase in NTHi in patients with very severe COPD compared to moderate COPD. Microbiome sequencing was conducted using 16S rRNA Illumina sequencing, V4 hypervariable region.	(Mayhew et al., 2018)
	Overall, there were significant microbiome shifts during AECOPD over one year, across moderate, severe and very severe COPD patients, between stable and exacerbation states. NTHi proliferation was associated with exacerbation events.	(Mayhew et al., 2018)
*Pseudomonas aeruginosa* causes AECOPD and prolongs hospitalisation.	Sputum culture and PCR analysis identified NTHi as most common bacteria (~24%) across 92 hospitalised AECOPD patients with mild (4.3%), moderate (19.5%), severe (10.8%) and very severe (65.2%) COPD (GOLD stage 1 – 4). However, NTHi was not significantly associated with hospitalisation duration.	(Nakou et al., 2014)
	*P. aeruginosa* was detected by culture as the second most common species across 92 patients hospitalised (~14%) and was significantly associated with increased hospitalisation duration.	(Nakou et al., 2014)
NTHi infections do not contribute to the COPD lung microbiome.	NTHi was not detected as part of the core microbiome in a small cohort of stable-state COPD patients. The microbiome was assessed using a terminal restriction fragment (TRF) length polymorphism and clone library analysis technique on bronchoalveolar lavage (BAL) samples from 9 stable state COPD patients with moderate (6/9) or severe (3/9) COPD (GOLD stage 2 - 3) compared to 9 healthy controls.	(Zakharkina et al., 2013)
NTHi infections do not contribute to AECOPD.	*Haemophilus* spp. were not identified as clinically important genera during AECOPD (0.7%). *Pseudomonas* spp. were also not clinically important (1.8%). In this study the sputum microbiota from a cohort of 9 patients with mild (1/9), moderate (3/9), and severe (5/9) COPD (GOLD stage 1 – 3) was analysed using 16S rRNA pyrosequencing and qPCR techniques.	(Jubinville et al., 2018)
*Moraxella* spp. and *Streptococcus* spp. contribute to severe AECOPD.	During exacerbation episodes, sputum samples from the 5 severe COPD patients (GOLD stage 3) showed increases of *M.* spp. and *S.* spp. in 90% and 88% patients, respectively.	(Jubinville et al., 2018)
AECOPD is associated with sputum microbiome shifts in *Proteobacteria, Firmicutes or Bacteroidetes.*	Between stable- and exacerbation-states, alpha-diversity analysis showed shifts in the proportion of Proteobacteria, Firmicutes and Bacteroidetes phyla. However, this dysbiosis was heterogeneous across patients.	(Jubinville et al., 2018)
*M. catarrhalis* infection is responsible for a subset of AECOPD but infection is cleared following immune response.	*M. catarrhalis* is estimated to cause 10% AECOPD. *M. catarrhalis* was detected by sputum culture in a prospective cohort study involving 104 COPD patients with 3009 clinic visits over 81 months. 560 visits occurred during exacerbation episodes and 2449 visits occurred during clinically stable periods. *M. catarrhalis* was detected in the sputum of 50 patients with 47.5% presenting with AECOPD. Immunoassays showed that patients cleared *M. catarrhalis* infections efficiently and molecular typing techniques showed that reacquisition of the same strain was rare, demonstrating development of strain-specific protection.	(Murphy et al., 2005)
Interspecific co-colonisation interactions exist in COPD patients.	A prospective cohort study involving monthly sputum cultures from 181 COPD patients exhibiting chronic bronchitis with 8843 clinic visits over 4.5 years revealed NTHi was the most common bacteria isolated (14.4%) and colonisation was positively correlated with *S. pneumoniae.* Co-colonisation correlation was consistent between stable-state and exacerbation states.	(Jacobs et al., 2018)
Lung tissue microbiota shifts in very severe COPD patients compared to smoker and non-smoker controls.	There is a reduction in diversity in severe COPD patients which also correlates with alveolar destruction. Bacterial DNA was isolated from lung tissue from 8 very severe COPD (GOLD stage 4) patients undergoing lung transplantation. Bacterial communities were analysed using qPCR amplification of 16S rRNA hypervariable region V2 and terminal restriction fragment length polymorphism analysis and pyrotag sequencing.	(Sze et al., 2012)
Bronchial wash microbiota shifts in COPD patients compared to smoker and non-smoker controls.	COPD is associated with a reduction in microbial diversity compared to smoking and non-smoking healthy controls, highlighting a microbial cause for COPD. The microbiome of bronchial wash samples in 18 clinically stable COPD patients with mild to severe (GOLD 1 – 3) airflow obstruction was significantly different to 8 healthy smokers and 3 non-smoker controls, detected by culture and Illumina MiSeq sequencing.	(Einarsson et al., 2016)
Lung microbiota does not shift during AECOPD and there does not account for exacerbation events.	Whilst dominant bacteria cultured from COPD patient sputum have included *P. aeruginosa* and *H. influenzae*, there were no significant microbiota changes before and after exacerbations. Overall microbial load and community composition remained stable following antibiotic treatment for AECOPD. Microbiota was analysed using anaerobic culture and 16S rDNA pyrosequencing of sputum from 40 patients with mild (17/40), moderate (17/40) or severe (6/40) COPD (GOLD stage 1 – 3).	(Tunney et al., 2013)
	In a longitudinal study analysing the microbiome of 476 sputum samples from 87 patients with mild (1/87), moderate (35/87), severe (32/87) and very severe (19/87) COPD (GOLD stage 1 – 4) patients, *Streptococcus, Haemophilus, Moraxella and Pseudomonas* accounted for 41.1%, 18.9%, 5.6% and 4.4%, respectively, of the total 366 genera and showed no statistically significant differences in composition before and during exacerbation. Sputum microbiota was analysed 16S rRNA pyrosequencing using the V3-V5 hypervariable region.	(Wang et al., 2016)
Dominant COPD lung microbiota are shared with healthy lung microbiota.	PCR amplification and 16S rRNA pyrosequencing of stable state sputum, bronchial aspirate, bronchoalveolar lavage and bronchial mucosa samples from 8 patients with moderate COPD (GOLD stage 3) showed 60% of the microbiota was dominated by genera shared with the healthy lung microbiota, including *Streptococcus*, *Prevotella*, *Moraxella*, *Haemophilus*, *Acinetobacter*, *Fusobacterium*, and *Neisseria *.	(Cabrera-Rubio et al., 2012)

GOLD, Global Initiative for Chronic Obstructive Lung Disease.

The general consensus reports that the healthy lung microbiota is largely comprised of *Pseudomonas, Streptococcus, Prevotella, Fusobacterium, Veillonella Porphyromonas and Haemophilus* spp. (Erb-Downward et al., 2011; Beck et al., 2012) that together represent >85% of the core lung microbiome. These genera have an important commensal role in modulating the immune response mechanisms of the lung and priming of the immune system (Segal et al., 2014). These genera are ubiquitous inhabitants of the lung microenvironment in both health and disease such as COPD, which is associated with an overall reduction in this microbial diversity. However, this microbial community composition remains diverse between healthy, non-smoker individuals, and microbial diversity further varies in healthy-smokers and COPD patients. Microbiota composition changes also occur in COPD patients as a consequence of the chronic infection process, host immune system responses and long-term use of bronchodilators, antibiotics and corticosteroid therapy (Toraldo and Conte, 2019). Previously, significantly different clusters of microbial communities have been reported between severe (GOLD 3/4) and less severe (GOLD 1/2) COPD patients (Mika et al., 2018), with correlations also being drawn between the abundance of Moraxellaceae and Streptococcaceae with the expression of immune system factors including IL-10 and TNF-α (Mika et al., 2018). Lung microbiome comparisons also detected significant increases in Proteobacteria and *Haemophilus* spp. and reductions in Bacteroidetes, *Prevotella* and *Veillonella* (Mayhew et al., 2018) in severe and very severe COPD patients compared to moderate COPD patients (Mayhew et al., 2018). Coupled with a decrease in overall diversity, these studies highlight how the lung microbiota increasingly destabilises throughout COPD progression.

Whilst this core, heterogeneous microbial diversity exists without necessarily causing disease, individually, all of these genera are also associated with microbial diversity shifts, or dysbiosis, that contribute to oral and airways disease states. These include periodontitis, cystic fibrosis, pneumonia, and inflammatory lung diseases (Pragman et al., 2016). The normal lung microbiome is extremely diverse, and some studies report *Neisseria* (Hilty et al., 2010), *Acinetobacter* (Cabrera-Rubio et al., 2012)*, Sphingomonas, Megasphaera* and *Staphylococcus* (Zakharkina et al., 2013) as predominant genera in both health and disease. Additionally, fungi, bacteriophages and viruses, including human rhinovirus (HRV), (Molyneaux et al., 2013) constitute part of this microbiome and have complex interactions with the bacteria, driving dysbiosis such as significantly increasing the proportion of Proteobacteria including *Haemophilus* spp. in the sputum microbiome of COPD patients (Molyneaux et al., 2013). Therefore, the existence of these bacteria in individuals in both health and disease states suggests that the aetiological factor for disease is not solely due to the presence of pathogens but a change in how those pathogens interact with the host and other bacteria i.e. their behaviour.

Of these genera, there is a substantial amount of evidence that suggests non-typeable *Haemophilus influenzae* (NTHi) is responsible for a large subset of AECOPD hospitalisations (Nakou et al., 2014; Wilkinson et al., 2017; Jubinville et al., 2018), alongside *Streptococcus pneumoniae* and *Moraxella catarrhalis* in several recent studies (**Table 1**). NTHi is an ubiquitous, non-encapsulated Gram-negative coccobacillus and opportunistic pathogen (Clementi and Murphy, 2011) despite having commensal properties (Hartwig et al., 2016). This commensal-turned-pathogen is the most common cause of *Haemophilus* spp. infection across all ages (Slack, 2017), is present in 70% COPD patients (Wilkinson et al., 2017) and is associated with 24% of AECOPD hospitalisations (Nakou et al., 2014). Furthermore, higher bacterial loads of NTHi are correlated with more severe airway inflammation, more severe AECOPD and increased disease burden on the patient (King and Sharma, 2015). However, NTHi presence does not explain every incidence of AECOPD across different patient cohorts, raising the question of how the behaviour of NTHi has changed in these cases to cause a disease state. One current hypothesis is that in developed countries, the routine immunisation of infants with the conjugated *H. influenzae* type B (Hib) vaccine and prescription of COPD maintenance drugs (Van Eldere et al., 2014) (Mayhew et al., 2018) has contributed to lung microbiome dysbiosis that favours NTHi colonisation and provides immunological pressures for the selection of biofilm-forming NTHi strains (Foxwell et al., 1998). For example, selection for NTHi strains expressing different outer-membrane proteins (OMPs) has led to a change in behaviour, favouring attachment and colonisation of the airways epithelia (Foxwell et al., 1998; Post et al., 2014), implicated in COPD infections (Cerquetti and Giufre, 2016). Similarly, antibiotic and maintenance treatments have been shown to be largely ineffective in combating the microbial causes of COPD in the long term. Systematic reviews have revealed that regular, long-term prophylactic antibiotic prescription did not result in reduced hospitalisation or improvement in lung function nor mortality (Herath et al., 2018). In addition, long-term maintenance drugs, including inhalation of corticosteroids, has been found to increase sputum bacterial load (Contoli et al., 2017). Moreover, short-term 3-month treatments with either moxifloxacin, doxycycline or azithromycin antibiotics were all found to not significantly decrease airway bacterial load but instead promoted antibiotic resistance in all treatment groups (Brill et al., 2015). These observations demonstrate how changes in the behaviour of NTHi, driven by disease progression and treatment strategies, may promote the biofilm lifestyle, contributing to disease.

Many severe COPD patients are receiving ‘long-term’ treatment with macrolide antibiotics that exhibit secondary anti-inflammatory properties and immune-modulating effects (Yao et al., 2013; Qiu and Zhong, 2017). Meta-analysis of several COPD cohorts has shown that macrolide antibiotic treatment successfully decreases the frequency of AECOPD when prescribed over 6 – 12 months (Yao et al., 2013) by suppressing bacterial load and reducing inflammation (Kelly et al., 2018). However, macrolides exert no benefit in the short term over 3 months (Yao et al., 2013). These findings have been corroborated using low-dose, long-term macrolide therapy up to 12 months (Cao et al., 2019) and current guidelines indicate optimal usage over 6 – 12 months in patients who experience 3 or more AECOPD per year, are prescribed steroids and are hospitalised due to AECOPD at least once per year (Smith et al., 2020). However, these guidelines do not describe long-term treatment that exceeds 12-months. This presents an issue for patients living with a progressive and currently incurable respiratory disease. Long term macrolide therapy also presents several risks, with studies reporting non-fatal but adverse effects including gastrointestinal reactions, liver injury and ototoxicity (Yao et al., 2013). In addition, due to the increased risk of comorbidities, macrolide therapy has been reported as being unsuitable for the elderly (Cao et al., 2019; Watson and Wilkinson, 2021). Further systematic reviews have reported the increasing risk of these adverse events as well as macrolide antibiotic resistance (Cui et al., 2018). This resistance is achieved by multiple mechanisms including modification of macrolide target sequences and upregulation of efflux pumps that increase with prolonged use of macrolides over 12-months (Djamin et al., 2020). Despite the benefit of macrolides in reducing AECOPD, much of the supporting evidence is attributed to azithromycin and not wider macrolides (Kelly et al., 2018), and ‘long-term’ treatment is limited to only 12 months. Accompanied by the reported risks of serious adverse events and acquisition of antibiotic resistance, macrolide therapy does not represent a substantial solution to controlling the bacterial and inflammatory components of COPD.

It is also important to recognise the limitations of these previous lung microbiome studies. There was previously no standardisation of microbiome analysis, such as the use of different 16S rRNA hypervariable regions (Beck et al., 2012). Many studies contained only small, cross-sectional cohorts (Wang et al., 2016). Furthermore, unprotected specimen brushings or sputum samples have also been widely used, which may introduce oropharyngeal contamination (Pragman et al., 2018). Together, these factors may limit the accuracy with which the healthy lung microbiome is reported and how it differs in disease states over time. However, results highlighting the importance of NTHi are reciprocated across several COPD cohorts by PCR, culture (Nakou et al., 2014; Wilkinson et al., 2017) and 16S rRNA techniques (Mayhew et al., 2018), warranting further investigation of NTHi and this pathogen’s life cycle within the COPD lung.

Different microbiome identification techniques should also be taken into consideration to allow the fair comparative appraisal of results between studies. A major advantage of culture techniques is that media is often selective and enriched with supplements that promote the growth of fastidious organisms, allowing low-abundance organisms to be detected and cultured (Hiergeist et al., 2015), whilst also eliminating contaminants. Furthermore, culture methods can lead to useful downstream experimental models and assays including antimicrobial susceptibility testing. However, whilst culture methods are improving, it is low-throughput and a central dogma is that only around 1% bacteria are culturable (Martiny, 2019), and those that can be cultured are likely altered under the nutrient-rich growth conditions. In addition, culture techniques may also introduce bias, depending on which selective growth media are used, based upon the microorganisms that are expected to be present in the sample. Together, these limitations mean the composition of bacteria detected using culture may not be a valid representation of the lung microbiome. Whilst 16S rRNA sequencing does not detect fungi and viral constituents, its major advantage is its sensitivity to the detection of phenotypically aberrant, rarely isolated or unculturable bacteria, as well as novel pathogens (Clarridge, 2004). Advances in Illumina sequencing and bioinformatic approaches also allow high-throughput detection, analysis and quantification of entire microbiome communities from multiple samples. However, high 16S rRNA sequence similarities may make it difficult to distinguish beyond the genus level in some taxa. The analysis is also dependent on the choice of one of nine bacterial 16S hypervariable regions (V1 – V9) with no single hypervariable region exhibiting a different enough degree of sequence diversity to distinguish amongst all bacteria at the species level (Chakravorty et al., 2007). Whilst not a sterile environment, the lung microbiome does have extremely low biomass which increases its susceptibility to contaminant sequences during sample acquisition, processing and 16S rRNA sequencing (Zubiria-Barrera et al., 2020). This may also explain high variances between studies.

It is clear however, that NTHi presents a significant problem for a large number of COPD patients and their clinicians alike, being detected across culture and 16S rRNA techniques. These patient-cohort focused studies show that, despite widespread use of antibiotics, NTHi persist in the COPD lung and lead to chronic and recurrent infections, exhibiting extreme tolerance to antimicrobial immune system defences and pharmacological treatments as well as displaying synergistic or co-infection interactions with other pathogens. Whilst NTHi intracellular infection is a well-documented mode of persistence in COPD, by becoming internalised and surviving within host cells as a protected reservoir that facilitates recurrent infection (Clementi and Murphy, 2011), NTHi exhibits distinct adaptations that are typical hallmarks of the biofilm lifestyle (Bjarnsholt, 2013). These include biotic surface adherence, extreme tolerance and resistance to antibiotics and antimicrobials, persistence of sub-populations of bacteria following treatment leading to recurrent infection, extreme capacity for evading host antimicrobial immunological defences and complex interspecific or polymicrobial interactions (Bjarnsholt, 2013).

### Non-Typeable *Haemophilus influenzae* Is Adapted for the Biofilm Lifestyle

**Figure 1:**
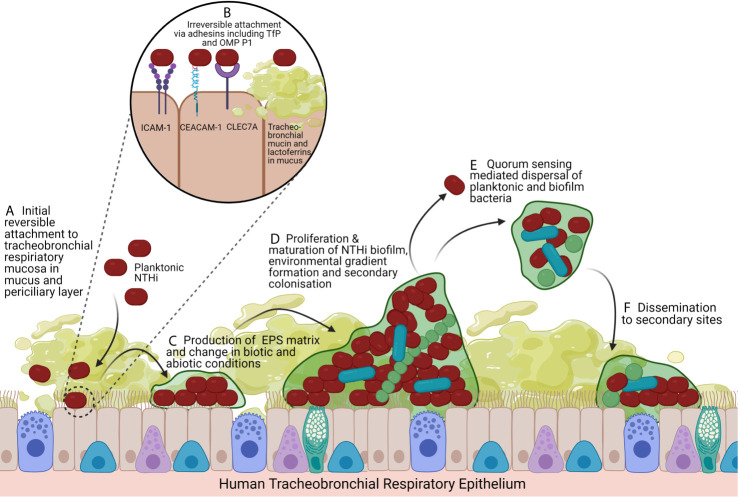
Model of NTHi Biofilm Formation and Lifecycle. **(A)** The impaired mucociliary clearance system coupled with inflammatory mucus secretion hyperplasia creates a suitable substrate for the initial colonisation and reversible attachment of NTHi to the air-liquid interface of the tracheobronchial respiratory mucosa, a pseudostratified, ciliated, columnar epithelium predominated by ciliated epithelial cells and interspersed by secretory cells (mucus-secreting goblet cells and secretoglobin secreting club cells) and basal cells. In COPD disease states, the mucus layer extends into the periciliary layer. **(B)** NTHi irreversibly attach to respiratory epithelial cells by adhesins including Type IV Pilus and OMP P1 that bind ICAM-1, CEACAM-1 and CLEC7A. NTHi also binds to the mucus layer itself by having adhesins that bind tracheobronchial mucins and lactoferrins. **(C)** Irreversibly attached NTHi begin to aggregate and produce an EPS matrix, favouring secondary colonisers. **(D)** Biofilms exhibit complex intraspecific and interspecific interactions, signalling, metabolism changes, gene expression changes that drive nutrient and oxygen gradients. This leading to differentiation of bacteria and maturation of the biofilm with structurally large microcolonies with nutrient and water channels. **(E)** Quorum sensing changes drive dispersal of the biofilm as planktonic bacteria and aggregates of biofilm leading to **(F)** dissemination and colonisation of secondary sites, facilitating persistence. Created with BioRender.com.

NTHi is well adapted for colonisation of the human airways. This fastidious organism survives in a mucus-rich environment and has a complex nutritional requirement for haemin and nicotinamide adenine dinucleotide, that are both available in the human lungs (Sriram et al., 2018). NTHi expresses an arsenal of proteins that allow it to adhere to and invade respiratory epithelial cells. These proteins include type IV pilus protein and the adhesin OMP P1 that bind to the host cell-surface protein ICAM-1 and glycoprotein CEACAM1 (Novotny and Bakaletz, 2013; Tchoupa et al., 2015), as well as mucin proteins and lactoferrins in human mucus (Kubiet and Ramphal, 1995). These mucus secretions and plugs that coat the airways epithelium may be especially important for the initial attachment stage in the development of bacterial biofilms (Langereis and Hermans, 2013) (**Figure 1**), which are sessile, three-dimensional multicellular communities of bacteria embedded within a self-produced extracellular polymeric substance (EPS) matrix (Costerton and Lewandowski, 1995). Biofilms exhibit complex strategies that confer persistent or chronic infection and dissemination to secondary sites of infection, characterised by tolerance and resistance to the immune system and antibiotic drugs.

## NTHi Biofilm Strategies

**Figure 2:**
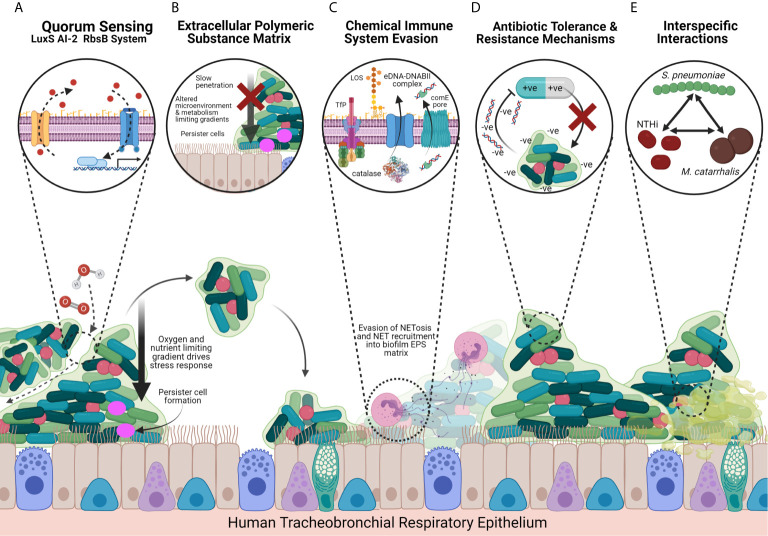
Model of NTHi Biofilm Strategies and Host-Pathogen Interactions Facilitating Chronic Infection in the COPD Airways. **(A)** The LuxS-RbsB AI-2 quorum sensing system facilitates coordination of intra-specific and inter-specific gene expression that promotes a transition from the planktonic to biofilm lifestyle in response to the local environmental conditions, leading to the downstream transcription of biofilm-associate genes. NTHi also express a QSeB two-component secondary system that may support biofilm gene expression coordination where the LuxS-RbsB system has been impaired. The quorum sensing system drives dissemination of biofilm bacteria to secondary sites, and formation of oxygen, nutrient and water channels towards the centre of the biofilm. **(B)** NTHi biofilms produce a thick protein- and eDNA-rich EPS matrix that provides a physical and chemical barrier, slowing penetration against pharmacological and immune system antimicrobial agents by blocking phagocytes and macrophages and chelating peptides and antibiotics such as β-defensin and β-lactams. The EPS matrix also provides structural stability and provides a site for mature biofilm structures to form, whilst also limiting metabolic activity, altering microenvironments, and driving persister cells through the development of gradients towards the centre of the biofilm. **(C)** NTHi express chemical defence strategies that allow for the evasion of the host’s NETosis response through recruitment of NETs into the EPS and expression of catalase that provides oxidative stress tolerance. Recognition by the immune system is also modulated through the differential expression of LOS and OMP P5 and OMP P2 that recruit neutrophils to form NETs but by evading the phagocytosing and cytotoxic mechanisms. NTHi biofilms further undergo a series of surface modifications that promote irreversible attachment, recruit secondary colonisers and impair recognition and/or clearance by immune system factors. These include sialyation, phosphorycholination, and the expression of TfP, OMPs and HMWs. **(D)** NTHi biofilms exhibit multidrug resistance due to upregulated carbohydrate metabolism and a preference for glycogen metabolism, as well as expressing antibiotic resistance genes including** **penicillin-binding protein 3 (PBP3) and β-lactamases. Negatively charged eDNA in the EPS matrix also sequesters positively charged antimicrobials. **(E)** Biofilms are seldom mono-species and exhibit complex inter-specific interactions with other inhabitants of the microbiome. In particular NTHi interacts with other COPD-associated pathogens including *S. pneumoniae* and *M. catarrhalis* that selects for hypoxia tolerant NTHi and damages cilia and mucociliary clearance, respectively, favouring NTHi biofilm formation. Tissue shown is the tracheobronchial respiratory mucosa, a pseudostratified, ciliated, columnar epithelium predominated by ciliated epithelial cells and interspersed by secretory cells (mucus-secreting goblet cells and secretoglobin secreting club cells) and basal cells. Created with BioRender.com.

The biofilm lifestyle confers several advantages to survival compared to the planktonic or free-living state (**Figure 2**). Typically, biofilm bacteria exhibit pleiomorphic behaviour, altering their morphology, coordinating gene expression and differentiating metabolomic functions in a heterogeneous manner. These changes occur in response to many factors such as: bacteria population density and quorum sensing, that regulates gene expression cascades; stressful environmental conditions, such as oxygen and nutrient gradients, that develop as the biofilm matures; the immune system; and pharmacological antimicrobial effectors. Additionally, biofilms exhibit physical and chemical strategies against the immune system through the formation of a protective barrier, the EPS matrix, as well as virulence factor modulation, interspecific synergistic interactions with other bacteria and the sharing of antimicrobial-sequestering compounds. The differentiation of metabolically inactive persister cells and dissemination of biofilm to secondary sites are major causes of chronic, recurrent infection following antibiotic treatment of the initial infection, which may be implicated in COPD.

### Quorum Sensing

Quorum sensing (QS) is the density-dependent coordination of gene expression (Costerton and Lewandowski, 1995; Langereis and Hermans, 2013) across bacteria within a community that is integral for biofilm formation, development and dispersal. Most biofilms achieve QS through autoinducer-2 (AI-2) chemical signalling (Langereis and Hermans, 2013). Autoinducer expression is encoded by the *LuxS* homologue (Armbruster et al., 2009) and these signals are detected by the ABC transporter RbsB (Armbruster et al., 2011), leading to further downstream transcription cascades that drive biofilm formation (**Figure 2**). This signalling mechanism highlights a target for interrupting biofilm development.

Like most other biofilm-forming bacteria, AI expression has been shown to promote NTHi biofilm formation and persistence, although much of this research is in the context of NTHi otitis media (OM) strains grown *in-vitro* (Armbruster et al., 2009) or the chinchilla middle ear model of NTHi-induced OM (Hong et al., 2009). Scanning confocal laser microscopy (SCLM) of NTHi *LuxS* mutants grown in continuous flow chambers show significantly reduced biomass, thickness and persistence (Armbruster et al., 2009). Disruption of the NTHi RbsB transporter reciprocated these results (Armbruster et al., 2011) showing that detection of QS signals is just as important as production. Downstream QS cascades result in increased transcription of biofilm-promoting genes including *gstA* – a glycosyltransferase that increases sialyation of the biofilm matrix (Pang et al., 2018), with *gstA* mutants producing sialyation deficient and low biomass biofilms.

However, NTHi also express a second, AI-2 independent QS system; the QSeB/C two component system that drives biofilm formation (Unal et al., 2012). However, QSeB/C disruption does not affect the biofilm in the same way as *LuxS* mutants. Instead, QSeB/C mutants show significant biofilm biomass and surface coverage in continuous flow models over 24 – 48 hours (Unal et al., 2012). Whilst this system is less well understood, the expression of a secondary QS system may explain why NTHi with a disrupted AI-2-RbsB system are still capable of forming reduced biofilms rather than no biofilms at all.

### Defence Against Immune System Effectors

Biofilms exhibit defence strategies against the immune system including survival and persistence within neutrophil extracellular traps (NETs) (**Figure 2**). NETs are extruding DNA-rich (Hong et al., 2009) webs of decondensed chromatin containing histones, neutrophil elastase and granules that trap bacteria (Dicker et al., 2018). One of the biofilm’s tolerance strategies is the expression of peroxiredoxin-glutaredoxin (pdgx) and catalase that protect bacteria against NET-derived oxidative stress effectors (Juneau et al., 2015) and expression is upregulated in biofilm compared to planktonic strains (Murphy et al., 2005). This NET-survival strategy has been demonstrated in NTHi biofilm OM models that form greater biomass over 14 days (Hong et al., 2009). Immunofluorescence and viability staining techniques demonstrated NTHi biofilms surviving and persisting within the NET lattices themselves. It is hypothesised that NETs can be incorporated into and benefit the structure of the biofilm (Hong et al., 2009). By increasing biomass, biofilms drive metabolism-limiting gradients that induce metabolically inactive persister cells at the centre of the biofilm and provide a physical and chemical barrier to immune system effectors and antimicrobial agents (Langereis and Hermans, 2013).

One of the lung’s primary defences against bacteria is activation of complement that opsonises invading pathogens for phagocytosis by neutrophils and macrophages (Ahearn et al., 2017). However, NTHi have adapted to evade or even ‘hijack’ the complement system, driving collateral damage and host tissue destruction (Su et al., 2018; Santocki and Kolaczkowska, 2020). Several studies report up- and down-regulation of virulence and complement activating factors including lipoligosaccharide (endotoxin) (West-Barnette et al., 2006), sialic acid, outer membrane proteins (OMP P2 and P5) and inflammatory mediators (King and Sharma, 2015) that trigger a local neutrophil response (**Figure 2**). However, because some NTHi have adapted to hijack complement and are inherently tolerant to NETosis, this increased neutrophil response instead promotes airways inflammation, facilitating intracellular invasion and persistent infection by NTHi.

### Extracellular Polymeric Substance: Key Components and Functions

The EPS matrix is a hallmark of biofilms which confers a protective barrier against the immune system and antimicrobials and is comprised of polysaccharides, proteins, lipids and eDNA (**Figure 2**), that may constitute 90% of the dry mass of most biofilms (Goodman et al., 2011). Until recently there was little evidence for the production of an EPS matrix (Langereis and Hermans, 2013) as part of the NTHi biomass. Unlike the polysaccharide-rich and alginate-producing biofilms of *P. aeruginosa*, the aetiological pathogen in chronic cystic fibrosis (CF) infections, NTHi biofilms have a limited carbohydrate or exopolysaccharide component (Domenech et al., 2016), containing only lipooligosaccharide (LOS) endotoxin. The composition of NTHi EPS is largely DNA and protein-rich which may contribute to its success in the hostile lung environment and tolerance to pharmaceuticals through a cascade of host-pathogen interactions (Gunn et al., 2016), despite not being embedded in a thick alginate.

Analysis of 18 biofilm-specific multifunctional proteins has demonstrated that the protein constituent of the EPS is essential for NTHi biofilm formation, maintenance, structural integrity, survival and tolerance to immune-system and antimicrobial effectors (Wu et al., 2014; Domenech et al., 2016). The EPS is structurally stabilised by Nuclear Associated Proteins DNABII that binds bent double stranded extracellular DNA (ds-eDNA), forming a strong nucleoprotein mesh (Goodman et al., 2011). Bent ds-eDNA are curved rather than straight architectures of extracellular DNA for which DNABII family proteins have a higher affinity (Goodman et al., 2011). This eDNA is independent of DNA derived from cell lysis or outer membrane vesicle biogenesis and instead NTHi employs an active mechanism of eDNA release *via* Tra-dependent inner membrane transit and release through the comE pore (Jurcisek et al., 2017). Bent ds-eDNA-DNABII complexes confer amoxicillin tolerance and increased biomass that is relinquished through antibody degradation of DNABII (Goodman et al., 2011). Immunofluorescent techniques have shown that eDNA provides structural stabilisation, maintenance and expansion of the biofilm to secondary sites (Domenech et al., 2016) and it is arranged in a meshwork of fine strands and thicker, rope-like ds-eDNA structures (Jurcisek and Bakaletz, 2007). Additionally, the negative charge of eDNA sequesters positively charged antimicrobials through a cation chelating interaction, conferring biocide resistance, requiring 1000-fold increases in antibiotic concentration to kill biofilms compared to planktonic phenotypes (Jones et al., 2013). In the chinchilla OM model, eDNA was also found to reduce the activity of the antimicrobial defence peptide (AMP) Beta Defensin-3 (Cavaliere et al., 2014). DNase I mediated degradation of eDNA resulted in reduced biofilm formation and increased susceptibility to ampicillin and ciprofloxacin *in-vitro* (Izano et al., 2009). Degradation of eDNA also destabilised the strong biofilms formed by clinical NTHi isolates and decreased surface adhesion and biofilm formation in planktonic cultures (Cavaliere et al., 2014).

However, the NTHi biofilm itself has its own eDNA control, mediated by the endogenous nuclease Nuc (Cho et al., 2015). Nuc is thought to be under the control of the QS system, leading to structural remodelling of the biofilm such as the formation of water and nutrient channels (Cho et al., 2015). Low-level expression of endogenous Nuc in NTHi biofilms exerts a similar destructive effect on these discrete areas of the biofilm in a similar way to treatment with DNAse I, but with a 1,400-fold greater activity (Cho et al., 2015). The high expression present in planktonic NTHi likely contributes to maintenance of the planktonic lifestyle. A second mechanism of structural remodelling is expression of the transcription inhibitor ModA2 methyltransferase that decreases eDNA and DNABII expression (Brockman et al., 2018), destabilising the biofilm. Therefore, both Nuc and ModA2 may be considered therapeutic targets of biofilm disruption, by increasing expression to drive NTHi biofilm instability and cause a biofilm to planktonic transition, making the bacteria more susceptible to treatment.

### Surface Component Modification and Adhesion Molecules

The NTHi outer membrane is particularly protein-rich and the expression of key proteins, including type IV Pilus, OMP P1 and P5 and HMW1/2 adhesins, together facilitate initial attachment directly to the bronchial epithelium and mucin-rich mucus (Kubiet and Ramphal, 1995; Jurcisek and Bakaletz, 2007; Giufre et al., 2008; Novotny and Bakaletz, 2013; Jurcisek et al., 2017), as well as trafficking of the aforementioned eDNA and DNABII components (**Figure 2**). Whilst NTHi are rich in only one polysaccharide, lipooligosaccharide (LOS), there is a diverse number of LOS and cell-surface modifications expressed in NTHi that are consistent with biofilm-forming bacteria (Swords et al., 2004). These LOS modifications include sialyation and phosphorylcholination that together increase biofilm formation and reduce LOS endotoxin bioactivity, reducing the host’s innate immune response to these substances (Greiner et al., 2004; Swords et al., 2004; West-Barnette et al., 2006). These modifications summarised below (**Table 2**) may also serve as therapeutic targets as interfering with key biofilm proteins and adhesins shows great potential in disruption of initial biofilm formation and maturation, and thus increases the biofilm’s susceptibility to treatments.

**Table 2:** Summary of Key NTHi Surface Modifications.

Surface Component Modification	Function	Experimental Effects	Citation
Sialyation	Sialytransferases modify the LOS with environmentally available sialic acid.	Sialyated LOS glycoforms have increased biofilm formation and persistence in the rat lung model system *in-vivo.* NTHi grown in sialic acid deficient conditions and *siaB* (CMO-sialic acid synthetase) have reduced biofilm formation.	(Swords et al., 2004)
		NTHi increase LOS sialyation during planktonic to biofilm transition which promotes aggregation and further sialyation.	(Greiner et al., 2004)
Phosphorycholine (ChoP)	A hydrophilic fatty-acid that promotes initial attachment and reduces immune response to infection.	Increased ChoP glycoforms reduces LOS endotoxin bioactivity, reducing host innate response stimulation and evasion of the immune system.	(Swords et al., 2004; West-Barnette et al., 2006)
Type IV Pilus (TfP)	Filamentous structure, 6-7nm in diameter common across many bacteria cell surfaces.	Immunofluorescent studies have revealed that TfP binds to human bronchial epithelia ICAM-1 receptor facilitating non-reversible attachment.	(Novotny and Bakaletz, 2013)
	In NTHi, encoded by PilA and transported to the cell membrane by ComE secretin in NTHi, contributing to biofilm formation.	In otitis media models, TfP was visualised to be part of the integral structure of the ds-eDNA component of the EPS matrix, constituting a function in further stability.	(Jurcisek and Bakaletz, 2007)
	Initial attachment by breaking through the substratum’s repulsive forces and forming weak but attractive van der Waals forces.	TfP machinery facilitates eDNA and DNABII trafficking and release into EPS matrix, *via* ComE pore where TfP is expressed.	(Jurcisek et al., 2017)
OMP P5/P1	A fimbrial structure that binds to mucin, a constituent of mucus. Chronic bronchitis mucus hypersecretion provides ideal nutrient-rich substrate to bind.	OMP P5 facilitates NTHi adherence to human tracheobronchial mucin and lactoferrins, promoting biofilm formation in the human lung.	(Kubiet and Ramphal, 1995; Novotny and Bakaletz, 2013)
		16S rRNA sequencing revealed *Streptococcus pneumoniae* was a dominant lung taxa during AECOPD.	(Jurcisek and Bakaletz, 2007)
		A recent study has reported OMP P1 as the genuine e CEACAM-binding invasin of *H. influenzae* leading to attachment and internalization in the absence of OMP P5 expression.	(Tchoupa et al., 2015)
HMW1/2 adhesins	Support direct adhesion to the upper respiratory tract	Present in 80% of clinical NTHi isolates however the expression varies.	(Giufre et al., 2008)

### Antibiotic Resistance and Tolerance Mechanisms

NTHi biofilms exhibit complex chemical multi-drug resistant strategies (**Table 3**) against a variety of widely used antibiotics (Slinger et al., 2006). Clinically important antibiotics including ciprofloxacin, azithromycin, and amoxicillin were 100% effective in eliminating 28 NTHi OM planktonic isolates whilst only killing 68%, 57% and 4% of NTHi biofilms, respectively (Slinger et al., 2006). Furthermore, 7% of biofilms even survived combined rifampicin - ciprofloxacin treatment. NTHi biofilms are inherently tolerant to high concentrations of gentamycin (Starner et al., 2006) and erythromycin (Starner et al., 2008). This tolerance is achieved partly through upregulated carbohydrate metabolism which drives transformation into the metabolically inactive biofilm phenotype dependent on stored glycogen (Wu et al., 2014). This is a protective, epigenetic response to sub-minimum inhibitory concentrations (sub-MIC) of β-lactam antibiotics (Wu et al., 2014) that target and inhibit the growth of metabolically active bacteria. Other proteomic changes occur acting against reactive oxygen species and promoting a semi-dormant lifestyle, rendering immune system factors and β-lactam antibiotics ineffective (Post et al., 2014). The aforementioned eDNA-rich EPS matrix protects against a range of antimicrobials including chlorhexidine glucoronate (Izano et al., 2009) and antibiotics including ampicillin and ciprofloxacin (Cavaliere et al., 2014). However, NTHi biofilms are, counter-intuitively, susceptible to sub-inhibitory concentrations of the macrolide antibiotic, azithromycin (Starner et al., 2008), which exhibits effective antibiofilm properties not demonstrated by erythromycin or gentamycin (Starner et al., 2008), even against clinical NTHi strains resistant to a wide range of antibiotics. However, the efficacy of azithromycin may not be due to only its antibacterial properties in this context. Recently, azithromycin has proved popular and effective for treatment of lung infections possibly due to its secondary anti-inflammatory properties (Southern and Barker, 2004). The COPD lung is characterised by chronic inflammation, increasing the risk of recurrent infection following treatment. The use of an antibiotic such as azithromycin with secondary anti-inflammatory properties may help decrease the lung’s innate immune system that causes collateral tissue destruction. Additionally, azithromycin has been reported to antagonise the quorum sensing system in *P. aeruginosa*, leading to decreased biofilm forming capacity, diminished virulence and an impaired oxidative stress response (Nalca et al., 2006), offering one explanation as to why β-lactam–azithromycin combination therapy in a cohort of critical care community-acquired pneumonia (CAP) patients reduced mortality by ~20% (Wise et al., 2010).

**Table 3:** Summary of NTHi Biofilm Antibiotic Resistance & Tolerance Mechanisms.

Mechanism & Drug	Model/Experiment	Experimental Effects	Citation
Gentamycin tolerance	Clinical CF NTHi isolates adhered to human airway epithelia	Biofilms survived treatment with high concentration gentamycin (10 – 25µl).	(Starner et al., 2006)
Sub-MIC β-lactam antibiotics & carbohydrate metabolism	NTHi biofilms grown on airway epithelia	mRNA transcriptional changes showed an increase in carbohydrate metabolism gene expression in response to sub-MIC ampicillin and amoxicillin.	(Wu et al., 2014)
		A subset of five genes functioned in glycogen biosynthesis, a component of biofilm biomass, as well as in the secretion of type IV pilus involved in adhesion. Furthermore, this exposure was found to ‘prime’ biofilms against stronger antibiotics and make them less sensitive to cefuroxime.	(Wu et al., 2014)
eDNA-rich EPS matrix protects against antimicrobials and antibiotics	8 clinical NTHi isolate static biofilms *in-vitro*	Biofilms were largely not susceptible to a range of detergents, antiseptics and disinfectants including sodium dodecyl sulfate, ceptpyridinium chloride, povidone and widely used clinical and commercially available chlorhexidine glucoronate.	(Izano et al., 2009)
		Treatment with DNase I degrades the eDNA component of the protective EPS matrix, drove dispersal and led to significantly greater killing of the bacteria by these agents.	(Cavaliere et al., 2014)
		Findings were reciprocated with ampicillin and ciprofloxacin treatment.	(Cavaliere et al., 2014)
Low metabolic activity protects biofilms against β-lactam antibiotics	Metabolomic and proteomic analysis of 814 proteins across biofilm and planktonic strains revealed that 127 products were differentially expressed.	Generally, proteins involved in protein synthesis and energy metabolism, including cysteinyl-tRNA synthetase and aerobic respiration control protein ArcA respectively, were largely downregulated.	(Post et al., 2014)
		DNA metabolism proteins and co-factor binding proteins including NAD nucleosidase (involved in oxidative stress) and heme-binding protein A were upregulated. Downregulation of metabolomic proteins suggests that NTHi biofilms survive in a dormant state with decreased energy metabolism and protein synthesis (Post et al., 2014) which may make β-lactam antibiotics, that act by inhibiting bacterial cell wall peptidoglycan, ineffective.	
	

### Multispecies Interactions With *S. pneumonia* and *M. catarrhalis*

Many studies investigate the role of biofilm-phase bacteria in the context of mono-species biofilms. However, because the lung microbiome is polymicrobial (Erb-Downward et al., 2011; Beck et al., 2012; Wilkinson et al., 2017) this diversity may facilitate complex but poorly understood interspecific interactions between bacteria as well as viruses (Molyneaux et al., 2013). These interspecific interactions (**Figure 2**) may be indirect, through changing of both biotic and abiotic conditions that favour the proliferation of secondary colonisers in airways diseases, analogous to ecological succession (Khanolkar et al., 2020). For example, NTHi infection has been found to upregulate the pro-inflammatory responses that may drive wider microbial shifts and exacerbate COPD (Rotta Detto Loria et al., 2013; Staples et al., 2016) as well as upregulate MUC2 production, increasing airway obstruction and providing a viscous substrate for secondary bacterial colonisers (Zhang et al., 2020) over time. Alternatively these interactions may be more direct through the growth of multi-species biofilms (Kyd et al., 2016) that result in co-operative adhesion and stability of the biofilm structure. NTHi and *S. pneumoniae* multi-species biofilms, for example, together produce and share a single EPS that is constituted by type IV pilus, eDNA, LOS, QS signals and other proteins, carbohydrates, adhesins and transcription factors (Kyd et al., 2016) from both species. Whilst further research is required to provide direct evidence as to whether NTHi is a primary coloniser of the human respiratory tract in COPD, NTHi does express an arsenal of adhesins and invasins that facilitate initial attachment directly to bronchial epithelia and mucin-rich mucus (Kubiet and Ramphal, 1995; Jurcisek and Bakaletz, 2007; Giufre et al., 2008; Novotny and Bakaletz, 2013; Jurcisek et al., 2017). Additionally, NTHi can express factors that change the environment making subsequent colonisation by other bacteria more favourable. NTHi and *S. pneumoniae* often co-colonise the respiratory tracts of COPD patients (Jacobs et al., 2018) and interact synergistically, promoting initial attachment, biofilm formation and survival. These interactions include increased expression of virulence genes such as the *pilA* of type IV Pilus (TfP), a strong and flexible transmembrane filament that has diverse functional roles in pathogenicity. Other interactions include the sharing of β-lactamases and eDNA (Jacobs et al., 2018), and the release of biocidal H_2_O_2_ (Tikhomirova and Kidd, 2013) that provides a nutrient and DNA reservoir at the expense of killed bacterial cells and senescent host cells, supporting biofilm survival. Co-culture of NTHi and *S. pneumoniae* strains was found to significantly increase biofilm formation on cultured respiratory epithelial cells (Krishnamurthy and Kyd, 2014) compared to mono-species biofilms. Whilst these bacteria also interact competitively, competition may not be a negative for NTHi survival in the long term. One such example is that *S. pneumoniae* expresses bactericidal H_2_O_2_ (Bair and Campagnari, 2019) and stimulates neutrophils that together provide a selection pressure for ROS-tolerant and persistent strains of NTHi (Tikhomirova and Kidd, 2013).

*M. catarrhalis* is a major respiratory pathogen that colonises 5-32% of COPD patients and accounts for 10% of acute exacerbations (Murphy et al., 2005). *M. catarrhalis* infections usually clear after 40 days but can result in long term biotic changes that favour secondary colonisation of the airways by NTHi (Murphy et al., 2005) following initial *M. catarrhalis* infection. *M. catarrhalis* binds to the tips of healthy ciliated epithelia and forms aggregates, significantly reducing cilia beat frequency in bronchial epithelial cultures (Velkova et al., 2018) which results in impairment of the mucociliary clearance pathway and formation of mucus plugs which become colonised by NTHi. In OM models, *M. catarrhalis* has been demonstrated to stabilise NTHi - *S. pneumoniae - M. catarrhalis* polymicrobial biofilms by protecting NTHi from the bactericidal properties of *S. pneumoniae*, further promoting NTHi viability and persistence (Bair and Campagnari, 2019).

## Evidence for NTHi Biofilms in the COPD Lung

A plethora of studies has identified single key genes and transcriptional products associated with biofilm formation and persistence *in-vitro* and *in-vivo* in bronchial epithelial and OM models, using clinical NTHi isolates (**Table 4**). Whilst these studies provide good evidence that NTHi has the capacity to form biofilms under nutrient-rich conditions, it does not confirm whether they actually form stable, persistent biofilms within the human COPD lung. Recently, however, the ferret COPD model, involving chronic long-term exposure to cigarette smoke, has provided immunofluorescent and gene expression evidence for NTHi biofilm formation and persistence (Hunt et al., 2020). Here, smoke-exposed ferret lungs, which presented characteristic histological and immunological hallmarks of COPD, showed increased NTHi aggregation and the expression of four key biofilm-associated genes, *pdgX, luxS, dps* and *hktE*, that are together involved in growth, quorum sensing, environmental stress and oxidative stress tolerance (Hunt et al., 2020).

**Table 4:** Summary of Clinical Evidence for NTHi Biofilms (*ex-vivo*).

Evidence	Model/Experiment	Experimental Effects	References
Biofilm promoting adhesin genes	PCR analysis of 108 clinical NTHi strains for adhesin genes correlated with biofilm formatting *in-vitro*	Variability in the presence of key adhesin genes including hifA (22%), hmw (48%), hia (57%), hap (22%) and siaB (38%), of which hemagglutinating pili and Hia were significantly associated with increased biofilm forming capacity. Biofilm forming strains of NTHi were significantly more likely to be identified in patients with chronic (90%) rather than acute (63%) respiratory infections.	(Nishi et al., 2006)
TEM showing biofilm adhesion to cell surfaces	*Ex-vivo* BALF imaged using TEM	Analysis of BAL from CF patients has provided good evidence of NTHi biofilm attached to cell surfaces, using transition election microscopy techniques These clinical NTHi isolates also formed mature biofilms on cultured airway epithelial cells, showing microcolony formation and EPS production.	(Starner et al., 2006)
Biofilm NTHi isolates	61 patients hospitalised with lower respiratory tract infection patients. NTHi isolated and grown *in-vitro*	Claimed to have an association with NTHi biofilm formation and increased hospitalisation duration. However, the evidence of biofilm production was poor, with only 10% actually forming biofilms, the majority of which (80%) were weak biofilm producers with no strong biofilms produced. Additionally, biofilms were no more significantly resistant to antimicrobials than non-biofilms so did not display a major hallmark of biofilm formation.	(Martinez-Resendez et al., 2016)
Biofilm NTHi isolates	Sarcoidosis patient sputum samples	*Haemophilus* spp. was identified in both 31/37 healthy and 30/31 sarcoidosis patients, 67% of *H. influenzae* isolates formed biofilm, all of which were weak using the standard crystal violet assay and classification.	(Kosikowska et al., 2016)
Immune response to biofilm strategies	6 COPD patient derived NTHi isolates and 18 COPD patients for ELISA analysis	COPD patient derived NTHi strains express greater pdgx under biofilm than planktonic growth conditions. ELISA detects pdgx antibodies in 44.4% COPD patients’ respiratory tract by ELISA showing host immune response to biofilm-survival strategies.	(Murphy et al., 2005)

Whilst recent reviews have highlighted several mechanisms of NTHi that favor epithelial cell adherence and biofilm formation in the lower airways (Short et al., 2021), there remains a gap in the research pertaining to NTHi biofilm development in the context of the COPD lung of which there is limited direct evidence (Ahearn et al., 2017). Some of the major limits to our understanding can be attributed to current methods of biofilm detection, which include isolation of NTHi from sputum and BAL samples and culture under nutrient-rich conditions *in-vitro* or non-lung *in-vivo* models.

Recently, PCR-based direct gene expression studies have revealed associations between adhesin expression and *in-vitro* biofilm formation, providing the first set of NTHi biofilm gene biomarkers (Nishi et al., 2006). However, this sub-set of genes is limited as thus far a gene expression profile using a comprehensive array of biofilm genes in NTHi clinical lung-derived samples has not been completed. Moreover, current imaging methods of *ex-vivo* biofilms are limited to transmission electron microscopy (Starner et al., 2006). Some studies report poor evidence of biofilm formation and in one cohort of COPD patients only 10% of NTHi isolates formed biofilm whilst 80% of these were classified as weak biofilms (Martinez-Resendez et al., 2016). Similarly, in a cohort of pulmonary sarcoidosis patients, another airways disease characterised partly by inflammation, there was a reduction in biofilm-forming *Haemophilus* spp. isolates in disease compared to health (Kosikowska et al., 2016) using culture methods.

Another study provided evidence for the existence of NTHi biofilm in the human respiratory tract as well as host responses to the biofilm in a cohort of COPD patients (Murphy et al., 2005). NTHi strains isolated from the sputum of six COPD patients grew biofilms expressing significantly more abundant pdgx compared to planktonic cultures and were shown to elicit an immune response in 44.4% of a further 18 COPD patients through detection of pdgx antibodies by ELISA (Murphy et al., 2005). This increased association of pdgx expression with biofilm formation is likely due to the protective properties against reactive oxygen species and survival within NETs (Juneau et al., 2015) which may serve as a biomarker for NTHi biofilm infection.

Overall, this evidence points to the existence of NTHi biofilms in infections across airways diseases. However, at the current time explicit evidence is limited in COPD. Further understanding the role of NTHi biofilms in COPD may elucidate novel, specific, therapeutic targets that may improve the disease management of patients where otherwise COPD maintenance drugs and antibiotics are ineffective in the long term.

## Discussion – Clinical Implications of NTHi Biofilm Research

The current literature provides strong genetic and phenotypic evidence that NTHi are well-adapted to have the capacity to colonise the human lung and form biofilms on bronchial epithelia with impaired mucociliary clearance, characteristic of COPD amongst other diseases. NTHi isolates are highly heterogeneous and further express an arsenal of strategies that confer immune system evasion, antimicrobial tolerance, persistence and chronic infection, that make it a particularly challenging pathogen to clinically treat. Considering the role of NTHi infections in the context of biofilms and their physical and chemical properties may explain the differences between patient cohorts, and why airways diseases are characterised by chronic, recurring lung infections. However, the presence of NTHi is not synonymous with AECOPD, so perhaps it is not necessarily the presence of a particular pathogen but the phase in which bacteria are present, biofilm or planktonic. Conversely, whilst some studies report NTHi as a non-significant bacteria in AECOPD or hospitalisation, this does not rule out the potential historical role of NTHi colonisation in the airways and host-pathogen interactions leading up to the present time of study. It is also important to consider that NTHi may have commensal benefits for the host’s immune system. For example, intracellular NTHi infection of airway epithelia protects against respiratory syncytial virus (RSV) (Hartwig et al., 2016) through stimulation of immune effectors. Conversely, NTHi can take advantage of compromised immune systems following viral infection (Molyneaux et al., 2013).

Understanding COPD and NTHi infections in the context of biofilms has several clinical implications. Firstly, the potential to improve diagnosis; currently, physical symptoms of chronic bronchitis and emphysema are used in the clinical diagnosis of COPD, despite recent microbiome associations. Whilst bacteria are understood to have a significant impact on the progression of this disease, dysbiosis of the lung microbiome is not currently used but could be a biomarker for predicting early onset COPD. Secondly, improving AECOPD treatment; NTHi isolates are highly heterogeneous in terms of biofilm-forming, antimicrobial tolerance and immune system stimulation or evasion mechanisms so a ‘one-treatment-fits-all’ approach may not be appropriate. Understanding the underlying biofilm mechanisms may reveal several targets to disrupt biofilm integrity, harmful host-pathogen interactions and increase antimicrobial susceptibility. Recently, nitric oxide donors have been successful in triggering biofilm dispersal from β-lactam resistant cystic fibrosis *P. aeruginosa* isolates and improving the efficacy of antibiotics (Cai and Webb, 2020; Soren et al., 2020). Mucolytic drugs are currently widely prescribed by clinicians as an adjunctive therapy for COPD (Papi et al., 2020) and include erdosteine that has been shown to increase the efficacy of antibiotics against chronic respiratory infections (Dal Negro et al., 2018). The adhesins OMP P5 and TfP that facilitate initial attachment have been targeted for vaccine development to prevent biofilm formation, which was successful in evoking a protective immune response to NTHi in the OM model (Novotny and Bakaletz, 2013). The literature demonstrates that targeting biofilm components are an effective way to reduce biofilm formation and increase antimicrobial susceptibility *in-vitro*, including antibody-mediated DNABII degradation (Goodman et al., 2011) and DNase I mediated eDNA degradation (Izano et al., 2009) with a plethora of surface modifications and tolerance mechanisms identified as future targets for development. Being able to profile the genotype or transcriptome of clinical isolates for biofilm-genes may reveal strain-specific susceptibilities or drug-targets where airways infections could be treated with greater efficacy using a precision medicine approach.

Despite recent advancements, there is a lack of evidence that NTHi forms biofilms within the human COPD lung. The majority of the research investigating this biofilm concept involves isolated strains cultured under nutrient-rich conditions *in-vitro* or in OM or bronchial epithelial cell culture models – not within the hostile lung environment with a cascade of immunological responses and potentially nutrient-deprived and anaerobic microenvironments. To our knowledge, there are no current studies that have attempted to detect NTHi biofilm within sputum, BALF samples or tissue biopsies from COPD patients *ex-vivo* using PCR detection of biofilm-associated genes or immunofluorescence imaging techniques. This would provide direct evidence of biofilms existing within the COPD lung.

Additionally, microbiome studies have a great deal of disparity reporting the core lung microbiome. Different studies take sputum and BAL samples which are different lung niches and may account for some of this disparity in microbiota shifts and diversity changes during AECOPD. Disparity can also be attributed to having no standard methodology, including sampling and sequencing inconsistencies, for example using different 16S hypervariable regions or using Illumina or Pyrosequencing platforms, that may yield different results when quantifying the abundance of bacteria taxa.

Future research should be directed towards clinical COPD NTHi isolates and model development that better represent the lung microenvironment and polymicrobial communities, to understand the behaviour of NTHi during chronic airways infections. Developing a comprehensive list of ‘biotypes’ for NTHi strains by profiling biofilm-gene expression could reveal patient-strain specific biofilm mechanisms, susceptibilities and potential therapeutic targets. Understanding the complex interspecific and host-pathogen interactions and microbiota shifts over time would allow us to model disease progression and design effective, precision-medicine treatment strategies to improve the prognosis of COPD patients and slow down the progression of COPD by targeting the microbial cause.

Understanding the behaviour of NTHi as a biofilm-producing pathogen reveals a growing number of mechanisms that could be potentially targeted by therapeutics. Research in model systems has already shown that disruption of key biofilm systems, including LuxS quorum sensing, the eDNA – DNABII binding complex, expression of key adhesins and cell surface modifications, alters the NTHi biofilm phenotype and increases antimicrobial susceptibility (Giufre et al., 2008; Starner et al., 2008; Armbruster et al., 2009; Jones et al., 2013; Cavaliere et al., 2014). However, the challenge now is to alter these processes within the human lung. Increasing our understanding of how NTHi coordinates the expression of genes that control these biofilm- and survival-promoting processes is essential. Recently, the role of bacterial exosomes or outer membrane vesicles (OMVs) have been implicated in NTHi biofilms in the air-liquid-interface epithelium model (Ren et al., 2012). Gram negative bacteria, including NTHi, produce spherically bilayered OMVs of approximately 100-300nm in size by a process of outward blebbing from the bacteria outer membrane (Jan, 2017) and have multifunctional roles including supporting the biofilm lifestyle (Gunn et al., 2016). Such proposed functions include the shuttling of quorum-sensing molecules (Ren et al., 2012) as well as enrichment with key outer membrane proteins (OMPs) and LOS that have previously been associated with biofilm development (Winter and Barenkamp, 2017) as well as NET recruitment (Sharpe et al., 2011). NTHi OMVs also traffic cargo destined for the EPS matrix (Gunn et al., 2016), a hallmark feature of biofilms that confers physical and chemical survival against the immune system and antimicrobial drugs. As analogous structures of eukaryotic extracellular vesicles that deliver a cargo of DNA, RNA and miRNA (Rodrigues et al., 2018; Guiot et al., 2019), it is possible that NTHi biofilm-derived OMVs represent a novel target for the disruption of gene expression pathways controlling biofilm development and contributing to disease.

This review has highlighted several key questions that need exploring in the field. Firstly, how does the behaviour of biofilm-phase NTHi affect progression of COPD? Specifically, there is a need to conduct research into lung-derived NTHi biofilms and develop valid airways models. Furthering our understanding of host-biofilm interactions, such as the proinflammatory cascade, could lead to slowing the progression of COPD. Next, the NTHi biofilm itself. Are the biofilm-specific strategies of NTHi the reason why disease management drugs and antimicrobials are largely ineffective in the long term? Whilst current treatment strategies do help to alleviate AECOPD and slow the progression of COPD, infections remain recurrent throughout the chronic disease leading to increased hospitalisation and morbidity (Wedzicha and Wilkinson, 2006; Wilkinson T, 2006; Kong and Wilkinson, 2020). This leads us to hypothesise that NTHi biofilms present potential, novel therapeutic targets (Wilkinson et al., 2019). For example, there is potential for biofilm development to be heavily impaired by targeting the aforementioned NTHi biofilm strategies including the eDNA-DNABII binding complex, adhesins, outer membrane proteins, high molecular weight proteins, LOS modifications and biofilm-promoting gene expression pathways.

## Conclusion

NTHi is a major aetiological commensal-turned-pathogen implicated in COPD progression and AECOPD across several recent large-cohort patient COPD studies. However, reports differ on the prevalence of this ubiquitous organism across healthy and non-healthy individuals, the reported significance of this pathogen in the onset or exacerbation of disease, as well as treatment efficacy. NTHi are well adapted to colonisation of the respiratory epithelium and express many genes that promote the biofilm lifestyle, as well as proteins that confer immune system evasion, antibiotic tolerance, and metabolomic changes that drive survival, persistence and chronic, recurrent infections. Furthering our understanding of the mechanisms of NTHi colonisation in chronic lung infections in COPD patients in the context of biofilms is now essential and may help to explain these vast differences. Whilst several studies report that clinical NTHi isolates can express the key hallmarks of a biofilm, these are often in the context of *in-vivo* OM or *in-vitro* airways epithelia models under nutrient-rich conditions that poorly represent the human lung. Further research is required to characterise the biofilm-forming capacity of NTHi and identify the COPD lung host-pathogen interactions and anti-biofilm therapeutic targets, which could have a significant impact on the diagnosis and treatment of patients living with chronic airways diseases.

## Author Contributions

JW, conceptualization, investigation, literature searching, analysis, project administration, writing original draft, reviewing and editing, approval of final draft. KS, supervision, conceptualization, reviewing and editing, approval of final draft. CS, supervision, conceptualization, reviewing and editing, approval of final draft. AW, supervision, reviewing and editing, approval of final draft. TW, supervision, conceptualization, reviewing and editing, approval of final draft. All authors contributed to the article and approved the submitted version.

## Funding

This work was undertaken using a BBSRC iCASE PhD studentship (BB/T508135/1) awarded for JW’s doctoral studies.

## Conflict of Interest

KS reports grants from AstraZeneca, outside the conduct of the study; TW reports grants and personal fees from AstraZeneca, outside the conduct of the study; personal fees and other from MMH, grants and personal fees from GSK, grants and personal fees from AZ, personal fees from BI, grants and personal fees from Synairgen, outside the submitted work.

The remaining authors declare that the research was conducted in the absence of any commercial or financial relationships that could be construed as a potential conflict of interest.

## Publisher’s Note

All claims expressed in this article are solely those of the authors and do not necessarily represent those of their affiliated organizations, or those of the publisher, the editors and the reviewers. Any product that may be evaluated in this article, or claim that may be made by its manufacturer, is not guaranteed or endorsed by the publisher.
